# Sequential Events in the Irreversible Thermal Denaturation of Human Brain-Type Creatine Kinase by Spectroscopic Methods

**DOI:** 10.3390/ijms11072584

**Published:** 2010-06-25

**Authors:** Yan-Song Gao, Jing-Tan Su, Yong-Bin Yan

**Affiliations:** State Key Laboratory of Biomembrane and Membrane Biotechnology, School of Life Sciences, Tsinghua University, Beijing 100084, China

**Keywords:** differential scanning calorimetry, human brain-type creatine kinase, intrinsic fluorescence, stepwise transitions, thermal denaturation

## Abstract

The non-cooperative or sequential events which occur during protein thermal denaturation are closely correlated with protein folding, stability, and physiological functions. In this research, the sequential events of human brain-type creatine kinase (hBBCK) thermal denaturation were studied by differential scanning calorimetry (DSC), CD, and intrinsic fluorescence spectroscopy. DSC experiments revealed that the thermal denaturation of hBBCK was calorimetrically irreversible. The existence of several endothermic peaks suggested that the denaturation involved stepwise conformational changes, which were further verified by the discrepancy in the transition curves obtained from various spectroscopic probes. During heating, the disruption of the active site structure occurred prior to the secondary and tertiary structural changes. The thermal unfolding and aggregation of hBBCK was found to occur through sequential events. This is quite different from that of muscle-type CK (MMCK). The results herein suggest that BBCK and MMCK undergo quite dissimilar thermal unfolding pathways, although they are highly conserved in the primary and tertiary structures. A minor difference in structure might endow the isoenzymes dissimilar local stabilities in structure, which further contribute to isoenzyme-specific thermal stabilities.

## 1. Introduction

When denatured by chemical denaturants, the unfolding of small single-domain proteins is generally a “all” or “none” two-state process, while that of large multimeric or multi-domain proteins is usually a much more complex process that involves a hierarchy of structural transitions [1,2]. As for thermal denaturation, it is traditionally demonstrated by a two-state model for most proteins. The pioneering work by Sheraga and his co-authors has indicated that the thermal unfolding of bovine pancreatic ribonuclease A is a multi-step process, with a sequential melting of protein subdomains or segments [3–6]. Since then, the sequential events in protein thermal unfolding have been characterized in some model proteins by resolution-enhancing spectroscopic techniques, such as two-dimensional correlation spectroscopy [7–20]. The identification of transitions around body temperature suggests that stepwise thermal unfolding may not only play a role in protein thermal stability, but also correlate with the physiological functions of proteins [19–22].

**Figure 1.**
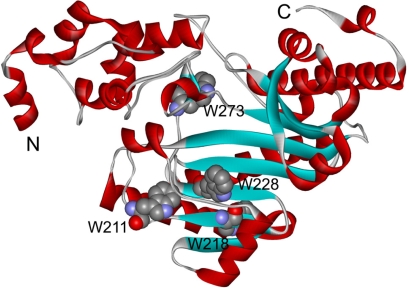
Structure of HBCK monomer (PDB ID: 3DRE) [27]. N and C denote the N- and C-terminus of the protein, respectively. The positions of the four Trp residues are highlighted by the space-filling model.

Creatine kinase (CK, EC 2.7.3.2) catalyzes the reversible phosphotransfer reaction between ATP/ADP and Cr/PCr. CK is proposed to be an “energy reservoir” that maintains intracellular ATP at a stable level and connects the different ATP subdomains in the excitable cells of higher vertebrates [23,24]. Two cytosolic isoforms with distinct tissue expression patterns have been identified for CK: the muscle-type (MMCK) and brain-type (BBCK) [25,26]. MMCK and BBCK are highly conserved in their sequence and share an almost superimposable tertiary structure [27]. The cytosolic CK exists as a dimer in solutions. Each subunit of CK is composed of two domains: a smaller N-terminal domain (NTD) containing only α-helices and a larger C-terminal domain (CTD) with both β-sheet and α-helix secondary structures (Figure 1). Since the first purification of rabbit muscle CK in 1954 [28], MMCK has been taken as a model enzyme and has been extensively studied in its structure, catalytic mechanism, physiological functions and folding, while only limited studies addressed the biophysical and biochemical properties of BBCK.

The folding mechanism of MMCK, when denatured or renatured in guanidine chloride or urea solutions, has been characterized as a multi-state process involving several intermediates [29–37]. Particularly, both kinetic and equilibrium folding studies have revealed the existence of a monomeric intermediate with unfolded NTD and folded CTD, and a dimeric inactive intermediate maintaining most of the native secondary structures. Both of these two intermediates are significantly disrupted at the tertiary structure level and are aggregation-prone, but the monomeric one is not accumulated during kinetic refolding [38]. The monomeric intermediate can be captured by the molecular chaperones GroEL and casein [39], while the dimeric intermediate can bind with the protein disulfide isomerase [38] and peptidyl-prolyl *cis-trans* isomerase [40].

The thermal denaturation of MMCK is dominated by a two-state irreversible process [41] with serious aggregation [42]. However, a pre-transitional conformational change in the CTD has been characterized by two-dimensional infrared spectroscopy and is proposed to be responsible for MMCK thermal aggregation [14]. The properties of the heat induced aggregation-prone state is similar to the dimeric inactivate intermediate that appeared during guanidine chloride denaturation, suggesting that the difference in the local stabilities of the CK molecule plays a crucial role in the onset of aggregation. However, no stable intermediate could be detected during the thermal denaturation of both rabbit and human MMCK [14,43].

Although MMCK and BBCK are highly similar in their structure and catalyze the same chemical reaction, isoenzyme-specific intracellular functions have been identified [44,45]. Recently, we also found that these two isoforms exhibit quite different properties during thermal inactivation [43]. The thermal stability of human MMCK (hMMCK) is much higher than that of human BBCK (hBBCK). The thermal inactivation of hMMCK is irreversible and is accompanied with serious aggregation, while that of hBBCK is partially reversible at temperatures below 55 °C. The distinct properties of these two isoenzymes suggest that they may undertake different structural changes during thermal denaturation. In this research, the sequential events in hBBCK thermal denaturation were investigated by spectroscopic methods.

## 2. Results and Discussion

### 2.1. Thermal Denaturation of hBBCK Is Calorimetrically Irreversible

**Figure 2.**
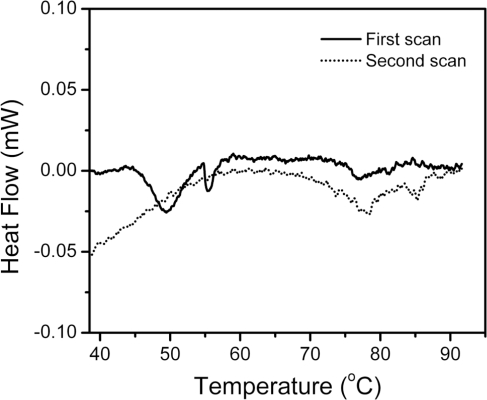
Temperature dependence of the heat flow of hBBCK thermal denaturation monitored by DSC. The heating rate was 1 K/min. The protein concentration was 1 mg/mL. The first scan is shown by the solid line, and the dotted line represents the second scan, which was performed by reheating the samples after cooling from the first scan. The negative peaks in the DSC profile are endothermic.

Our previous study has indicated that the thermal inactivation of hBBCK is partially reversible at temperatures below 55 °C, but no activity could be recovered by 24 h reactivation on ice for samples pre-heated at temperatures above 60 °C [43]. To elucidate whether the irreversibility was caused by the temperature-jump during cooling, differential scanning calorimetry (DSC) experiments were performed. As shown in Figure 2, the heat flow *versus* temperature profile contained several endothermic peaks, suggesting that the thermal denaturation of hBBCK contained stepwise transitions. The main peak was centered at about 50 °C, followed by a small peak at around 56 °C. At around 77 °C, there was an additional weak endothermic peak, which might be the further unfolding of the residual structures maintained in the non-native oligomers or small aggregates. It is noteworthy that no changes were observed in the turbidity of the solution when monitored by absorbance at 400 nm, even when the sample was heated up to 100 °C for 10 min, suggesting that the non-native oligomers characterized in our previous study [43] did not associate into large amorphous aggregates at extremely high temperatures. This behavior is quite different from the thermal denaturation of MMCK [14,41,42]. The second scan was performed by heating the solutions up to 92 °C, cooling them at 20 °C, and reheating them to collect the calorimetric profile. No obvious thermal effect was observed in the area of the main endothermic peaks (45–60 °C) during the first scan, implying that the denaturation of hBBCK induced by heat was mainly dominated by a calorimetrically irreversible process. It is worth noting that several peaks and knobs appeared at high temperatures for the second scan, which might be due to the further unfolding of the residual structures or the partial reversibility of the transitions at high temperatures, or both.

**Figure 3.**
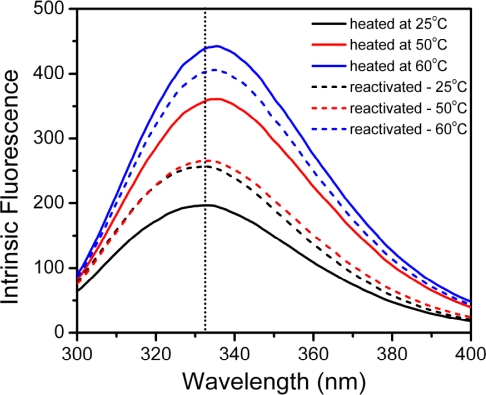
Intrinsic fluorescence spectra of the thermally inactivated and reactivated hBBCK samples. The inactivated samples were prepared by heating the enzyme solutions (0.2 mg/mL) at 25 °C, 50 °C, and 60 °C for 10 min, while the reactivated samples were prepared by incubating the inactivated samples on ice for 24 h. The intrinsic fluorescence was measured with an excitation wavelength of 280 nm.

To further study the reversibility of the hBBCK structure during thermal denaturation, intrinsic fluorescence spectra were collected for samples heated at a given temperature and reactivated on ice for 24 h. The typical spectra are presented in Figure 3. Since the intensity of the intrinsic fluorescence spectra of proteins is easily affected by many environmental factors, the maximum emission wavelength (*E*_max_) was used to reflect the structural integrity of the protein. After 24 h incubation, no significant change was observed in the *E*_max_ value as well as the shape of the spectrum of the samples heated at 25 °C. For samples heated at 50 °C and 60 °C, the intrinsic fluorescence spectra had a red-shift of about 3 nm after being reactivated for 0 h. After 24 h of reactivation, the *E*_max_ value of the sample pre-treated at 50 °C could recover to that of the native enzyme, whereas the sample pre-treated at 60 °C could not. These observations suggested that the irreversible inactivation of hBBCK at high temperatures observed in our previous research [43] was caused by the irreversible changes in hBBCK tertiary structure.

### 2.2. hBBCK Thermal Denaturation Monitored by CD Spectroscopy

**Figure 4.**
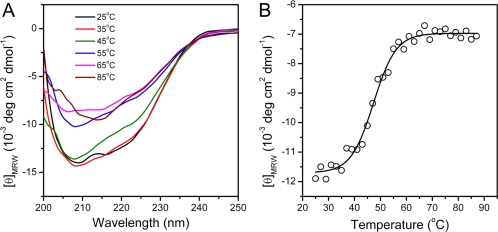
hBBCK thermal denaturation monitored by CD spectroscopy. (**A**) Typical CD spectra of hBBCK recorded at a given temperature after 2 min equilibration. (**B**) Changes in the ellipticity at 222 nm with the increase of temperature. The CD data are presented as mean residue molar ellipticity ([θ]_MRW_).

Far-UV CD spectroscopy is a sensitive monitor of changes in protein secondary structure [46]. The CD spectrum recorded at 25 °C exhibited a typical shape of α + β proteins (Figure 4), which is consistent with the structure of hBBCK shown in Figure 1. At high temperatures, the shape of the CD spectra revealed that denatured protein was dominated by β-sheet structures and lacked helical structures. This was reflected by the single negative peak at around 215 nm. The change in the CD data was best-fitted by a two-state process, and the midpoint temperature of transition (*T*_m_) was 47.3 ± 0.4 °C.

### 2.3. hBBCK Thermal Denaturation Monitored by Intrinsic Fluorescence Spectroscopy

**Figure 5.**
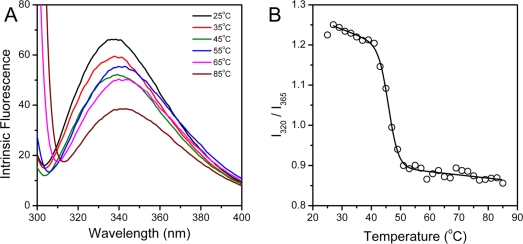
hBBCK thermal denaturation monitored by intrinsic fluorescence spectroscopy excited at 295 nm. (**A**) Fluorescence spectra of hBBCK recorded at the given temperature after 2 min equilibration. (**B**) Dependence of *I*_320_/*I*_365_ on temperature.

hBBCK contains four Trp residues located in the CTD (Figure 1), while the Tyr residues are distributed in both the NTD and the CTD. To probe the tertiary structural changes of hBBCK during thermal denaturation, intrinsic fluorescence were measured using an excitation wavelength of both 280 nm and 295 nm. When excited at 280 nm, the fluorescence is from the contributions of both Trp and Tyr, while only the Trp fluorophores are detected when excited at 295 nm. No significant difference in the transition curves was observed by the two excitation wavelengths, and thus the spectra excited at 295 nm were used for further analysis. As shown in Figure 5A, the Trp fluorescence had a slight red-shift (∼5 nm) during thermal denaturation. The relative small change made it difficult to measure the *E*_max_ value precisely. Thus *I*_320_/*I*_365_, which is a measure of the spectral shape [47], was used to reflect the change in the Trp fluorescence spectra during heating. The *I*_320_/*I*_365_ value decreased slightly when the temperature increased from 25 °C to 40 °C, suggesting that the fluorophores had become more water-accessible during heating. The main transition occurred in the temperature range of 41–51 °C with a *T*_m_ value of 45.7 ± 0.2 °C.

**Figure 6.**
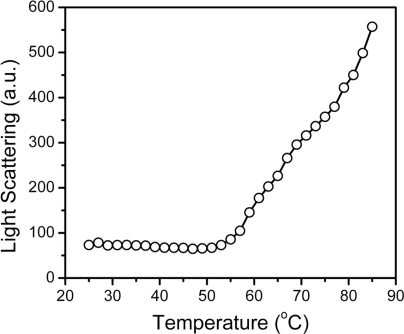
Changes in the oligomeric states during hBBCK thermal denaturation monitored by resonance light scattering.

During thermal denaturation, MMCK is prone to form large aggregates that are easily detected by turbidity measurements. Our previous study indicated that hBBCK only formed non-native oligomers or small aggregates that could not be detected by turbidity experiments. However, these aggregates are visible in the size-exclusion chromatography when quenched on ice. To monitor the formation of the oligomers during the heating process, resonance Rayleigh light scattering was monitored as a function of temperature (Figure 6). No significant changes in the light scattering occurred at temperatures below 51 °C, followed by a continuous increase for temperatures higher than 51 °C. Two transitions could be distinguished, as seen in Figure 6, with temperatures ranging from 51 °C to 77 °C and >77 °C, respectively. These transitions coincided with the existence of additional endothermic peaks at around 77 °C in the DSC profile (Figure 2).

**Figure 7.**
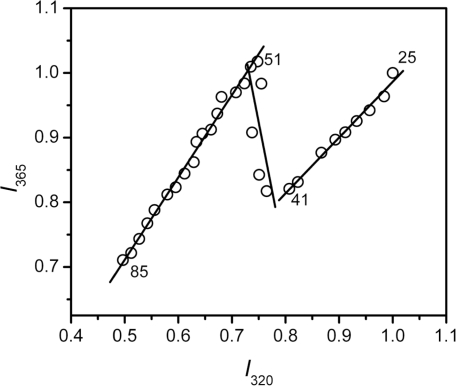
Phase diagram analysis of the fluorescence data shown in Figure 5. The diagram was constructed by plotting *I*_320_ *versus I*_365_. The *I*_320_ and *I*_365_ values were normalized by the maximum value in each set of the fluorescence data.

The *T*_m_ value from intrinsic fluorescence was about 1.5 °C smaller than that from CD spectroscopy, suggesting that the changes in hBBCK secondary and tertiary structures were not synchronous. To further probe the stepwise changes in hBBCK thermal denaturation, a phase diagram was constructed to detect the asynchronous changes of the hydrophobic (monitored by *I*_320_) and hydrophilic fluorophores (monitored by *I*_365_). The diagram of hBBCK thermal denaturation was composed of three linear parts with joint positions at 41 °C and 51 °C, respectively (Figure 7). In the phase diagram, each straight line represents a two-state process, and the joint position indicates the appearance of a possible intermediate [48]. Thus, the results in Figure 7 indicated that the microenvironmental changes of hBBCK Trp residues induced by heat involved at least three distinct transitions.

**Figure 8.**
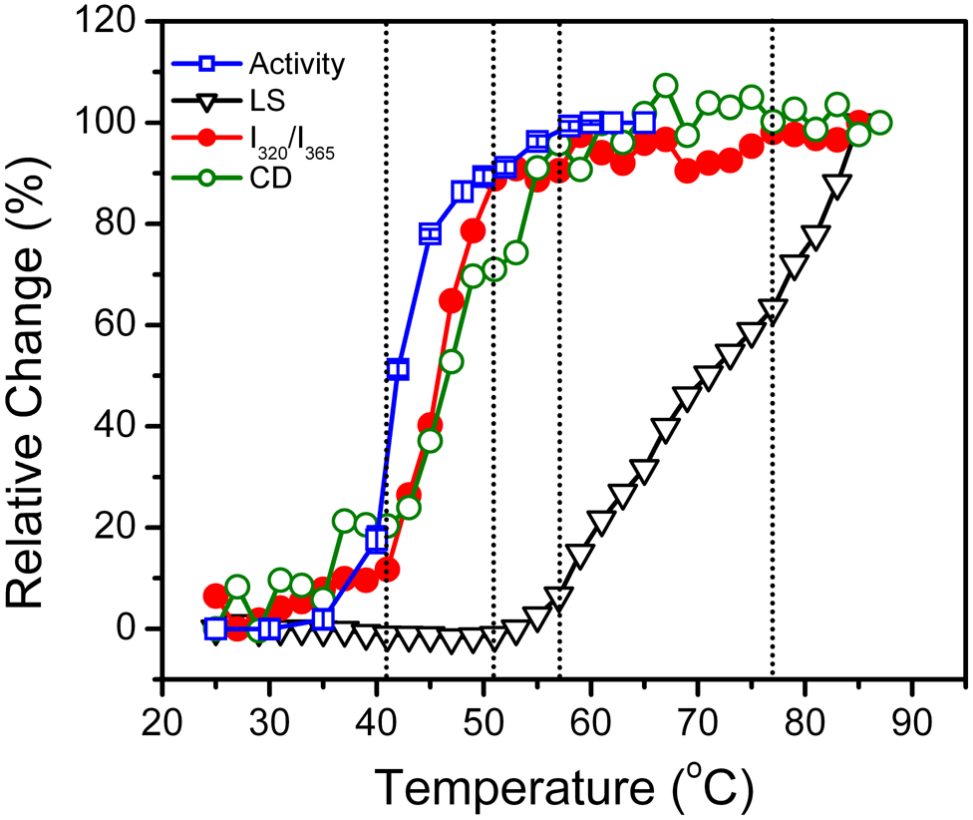
A summary of the transition curves obtained by various probes. The activity data were from [43].

The above results from spectroscopic analysis clearly indicated that the thermal denaturation of hBBCK was a stepwise process. A summary of the transition curves is presented in Figure 8. When temperature increased from room temperature to body temperature, no significant change was observed in hBBCK secondary structures. However, a minor increase was observed in the solvent exposure of the Trp fluorophores, which might reflect the increase of structural fluctuations that correspond with the increase of temperature. The enzyme began to lose its activity from 41 °C. This loss of activity was accompanied by significant structural changes at both the secondary and tertiary structure levels. The activity loss occurred prior to the structural transitions, suggesting that the modification of the active site structure proceeded from the unfolding of the overall structure. At 51 °C, hBBCK lost most of its activity and the changes in the microenvironments of the Trp fluorophores was finished, while a further decrease was found in the hBBCK regular secondary structures at temperatures above 51 °C. These asynchronous structural transitions might be the reason why two endothermic peaks were observed in the DSC profile within the temperature range of 40–60 °C. At temperatures above 55 °C, the unfolded molecules began to associate into non-native oligomers. An increase in either the associating rate or the size of the oligomers occurred at 77 °C, which was confirmed by the appearance of an additional endothermic peak centered at around 77 °C in the DSC profile. These sequential events were quite different from those of MMCK [14]. As for MMCK, the unfolding of native structures is closely correlated to thermal aggregation. In contrast to MMCK, the unfolding and aggregation were sequential or separated events for hBBCK. These results also suggested that the isoenzymes had differential local stabilities in their structure, which might contribute to the discrepancy in their thermal stabilities.

It is worth noting that both hMMCK and hBBCK were not fully unfolded during thermal denaturation. The changes of the *E*_max_ values, in particular, were minor for both proteins when compared to the chemical denaturants-induced unfolding. When denatured by guanidine chloride, the *E*_max_ of the fully unfolded state is at about 350 nm [32,49–51], which reflects that all of the Trp fluorophores are fully exposed to the solvent. However, the *E*_max_ of the thermally denatured state was at around 342 nm, implying that most of the fluorophores had limited solvent accessibility. These observations are consistent with the proposal that protein thermal aggregation involves the exposure of certain regions and does not require the protein to be fully unfolded [20].

## 3. Experimental Section

### 3.1. Materials

Sodium dodecylsulfate (SDS), isopropyl-1-thio-β-D-galactopyranoside (IPTG), Tris, creatine, ATP, DTT, and thymol blue were purchased from Sigma. All the other reagents were local products of analytical grade.

### 3.2. Protein Expression and Purification

Recombinant hBBCK was overexpressed in *Escherichia coli* BL21 [DE3]-pLysS (Stratagene, Germany) and purified as described previously [43,51]. The final products were collected from a Sephacryl S300 HR column equipped on an ÄKTA purifier. The protein concentration was determined according to the Bradford method by using bovine serum albumin as a standard [52]. All enzyme solutions were prepared by dissolving the proteins in 10 mM Tris-HCl at a pH of 8.0.

### 3.3. Activity Assay

CK activity was measured according to the pH-colorimetry method [29] in the phosphocreatine formation direction by monitoring the absorbance changes at 597 nm on an Ultraspec-4300 Pro spectrophotometer. All the activity experiments were performed at 25 °C and repeated for at least three times. The thermal inactivated and reactivated samples were prepared using the sample procedures as described previously [43].

### 3.4. DSC Experiments

DSC experiments were performed using a Setaram Micro DSC III calorimeter. The final protein concentration was 1 mg/mL for the DSC experiments, and the solution volume was 0.8 mL. The DSC profiles were obtained using a scan rate of 1 K/min from 20 °C to 92 °C. The second scan was carried out by reheating the samples after they were cooled to 20 °C and equilibrated for 30–40 min.

### 3.5. Spectroscopy

All resultant spectra were obtained by the subtraction of the control. Far-UV circular dichroism (CD) spectra were recorded on a Jasco-715 spectrophotometer using a cell with a path length of 0.1 cm. The formation of the non-native oligomers was monitored by measuring the resonance light scattering at 90° using Trp as the intrinsic fluorophore excited at 295 nm [53]. Intrinsic fluorescence spectra were measured on a Hitachi F2500 or F4500 spectrophotometer with an excitation wavelength of 280 nm (to excite both Trp and Tyr) or 295 nm (to excite Trp only). The phase diagram of the fluorescence data was constructed using the published procedure [48]. In brief, the fluorescence intensity at 320 nm (*I*_320_) and 365 nm (*I*_365_) were normalized by the maximum of each data set, and then the diagram was constructed by plotting *I*_320_ *versus I*_365_.

## 4. Conclusions

In this research, the sequential events in hBBCK thermal denaturation were studied by DSC, CD, and intrinsic fluorescence spectroscopy. The DSC results indicated that the thermal denaturation of hBBCK was calorimetrically irreversible. The discrepancy in the transition curves obtained from various probes suggested that hBBCK undergo sequential structural changes when subjected to thermal stress. During heating, the disruption of the active site structure occurred prior to the secondary and tertiary structural changes. The thermal unfolding and aggregation of hBBCK was found to occur via sequential events, which is quite different from that of MMCK. The results herein suggested that hBBCK and hMMCK undergo quite dissimilar thermal unfolding pathways, although they are highly conserved in their primary and tertiary structures. The minor difference in structure might endow the isoenzymes dissimilar local stabilities in structure, which further contribute to the isoenzyme-specific thermal stabilities.

## References

[1] Jaenicke R Stability and folding of domain proteins Prog. Biophys. Mol. Biol 1999 71 155 241 10097615 10.1016/s0079-6107(98)00032-7

[2] Jaenicke R Lilie H Matthews CR Folding and association of oligomeric and multimeric proteins Adv. Protein Chem 2000 53 329 362 10751948 10.1016/s0065-3233(00)53007-1

[3] Burgess AW Scheraga HA A hypothesis for the pathway of the thermally-induced unfolding of bovine pancreatic ribonuclease J. Theor. Biol 1975 53 403 420 560 10.1016/s0022-5193(75)80012-9

[4] Matheson RR Jr Scheraga HA Steps in the pathway of the thermal unfolding of ribonuclease A. A nonspecific photochemical surface-labeling study Biochemistry 1979 18 2437 2445 36132 10.1021/bi00579a001

[5] Matheson RR Jr Scheraga HA Steady-state kinetic study of action of ribonuclease A, involving a conformational change between 30 and 40 degrees C Biochemistry 1979 18 2446 2450 571735 10.1021/bi00579a002

[6] Navon A Ittah V Laity JH Scheraga HA Haas E Gussakovsky EE Local and long-range interactions in the thermal unfolding transition of bovine pancreatic ribonuclease A Biochemistry 2001 40 93 104 11141060 10.1021/bi001945w

[7] Stelea SD Keiderling TA Pretransitional structural changes in the thermal denaturation of ribonuclease S and S protein Biophys. J 2002 83 2259 2269 12324443 10.1016/S0006-3495(02)73986-6 PMC1302314

[8] Stelea SD Pancoska P Benight AS Keiderling TA Thermal unfolding of ribonuclease A in phosphate at neutral pH: Deviations from the two-state model Protein Sci 2001 10 970 978 11316877 10.1110/ps.47101 PMC2374205

[9] Vermeer AW Norde W The thermal stability of immunoglobulin: Unfolding and aggregation of a multi-domain protein Biophys. J 2000 78 394 404 10620303 10.1016/S0006-3495(00)76602-1 PMC1300647

[10] Paquet MJ Laviolette M Pezolet M Auger M Two-dimensional infrared correlation spectroscopy study of the aggregation of cytochrome c in the presence of dimyristoylphosphatidylglycerol Biophys. J 2001 81 305 312 11423415 10.1016/S0006-3495(01)75700-1 PMC1301512

[11] Dong A Randolph TW Carpenter JF Entrapping intermediates of thermal aggregation in ahelical proteins with low concentration of guanidine hydrochloride J. Biol. Chem 2000 275 27689 27693 10871628 10.1074/jbc.M005374200

[12] Fabian H Mantsch HH Schultz CP Two-dimensional IR correlation spectroscopy: Sequential events in the unfolding process of the l cro-V55C repressor protein Proc. Natl. Acad. Sci. USA 1999 96 13153 13158 10557289 10.1073/pnas.96.23.13153 PMC23916

[13] Yan Y-B Wang Q He H-W Hu X-Y Zhang R-Q Zhou H-M Two-dimensional infrared correlation spectroscopy study of sequential events in the heat-induced unfolding and aggregation process of myoglobin Biophys. J 2003 85 1959 1967 12944308 10.1016/S0006-3495(03)74623-2 PMC1303367

[14] He H-W Zhang J Zhou H-M Yan Y-B Conformational change in the C-terminal domain is responsible for the initiation of creatine kinase thermal aggregation Biophys. J 2005 89 2650 2658 16006628 10.1529/biophysj.105.066142 PMC1366765

[15] Zhang J Yan Y-B Probing conformational changes of proteins by quantitative second-derivative infrared spectroscopy Anal. Biochem 2005 340 89 98 15802134 10.1016/j.ab.2005.01.053

[16] Yan Y-B Zhang J He H-W Zhou H-M Oligomerization and aggregation of bovine pancreatic ribonuclease A: Characteristic events observed by FTIR spectroscopy Biophys. J 2006 90 2525 2533 16415060 10.1529/biophysj.105.071530 PMC1403177

[17] Zhang J He H-W Wang Q Yan Y-B Sequential events in ribonuclease A thermal unfolding characterized by two-dimensional infrared correlation spectroscopy Protein Pep. Lett 2006 13 33 40 10.2174/092986606774502027 16454667

[18] Su J-T Kim S-H Yan Y-B Dissecting the pretransitional conformational changes in aminoacylase I thermal denaturation Biophys. J 2007 92 578 587 17071653 10.1529/biophysj.106.093666 PMC1751394

[19] Stadler AM Digel I Artmann GM Embs JP Zaccai G Buldt G Hemoglobin dynamics in red blood cells: Correlation to body temperature Biophys. J 2008 95 5449 5461 18708462 10.1529/biophysj.108.138040 PMC2586580

[20] Yan Y-B Wang Q He H-W Zhou H-M Protein thermal aggregation involves distinct regions: Sequential events in the heat-induced unfolding and aggregation of hemoglobin Biophys. J 2004 86 1682 1690 14990496 10.1016/S0006-3495(04)74237-X PMC1304004

[21] Digel I Maggakis-Kelemen C Zerlin KF Linder P Kasischke N Kayser P Porst D Artmann AT Artmann GM Body temperature-related structural transitions of monotremal and human hemoglobin Biophys. J 2006 91 3014 3021 16844747 10.1529/biophysj.106.087809 PMC1578488

[22] Zerlin KFT Kasischke N Digel I Maggakis-Kelemen C Artmann AT Porst D Kayser P Linder P Artmann GM Structural transition temperature of hemoglobins correlates with species’ body temperature Eur. Biophys. J 2007 37 1 10 17390129 10.1007/s00249-007-0144-4

[23] Wallimann T Wyss M Brdiczka D Nicolay K Eppenberger HM Intracellular compartmentation, structure and function of creatine kinase isoenzymes in tissues with high and fluctuating energy demands: The ‘phosphocreatine circuit’ for cellular energy homeostasis Biochem. J 1992 281 21 40 1731757 10.1042/bj2810021 PMC1130636

[24] Wallimann T Bioenergetics: Dissecting the role of creatine kinase Curr. Biol 1994 4 42 46 7922310 10.1016/s0960-9822(00)00008-7

[25] Wallimann T Hemmer W Creatine-kinase in nonmuscle tissues and cells Mol. Cell. Biochem 1994 133 193 220 7808454 10.1007/BF01267955

[26] McLeish MJ Kenyon GL Relating structure to mechanism in creatine kinase Crit. Rev. Biochem. Mol. Biol 2005 40 1 20 15804623 10.1080/10409230590918577

[27] Bong SM Moon JH Nam KH Lee KS Chi YM Hwang KY Structural studies of human brain-type creatine kinase complexed with the ADP-Mg^2+^-NO_3_-creatine transition-state analogue complex FEBS Lett 2008 582 3959 3965 18977227 10.1016/j.febslet.2008.10.039

[28] Kuby SA Noda L Lardy HA Adenosinetriphosphate-creatine transphosphorylase. I. Isolation of the crystalline enzyme from rabbit muscle J. Biol. Chem 1954 209 191 201 13192073

[29] Yao QZ Zhou HM Hou LX Zou CG A comparison of denaturation and inactivation rates of creatine kinase in guanidine solutions Sci. Sin. B 1982 25 1296 1802 7167806

[30] Morris GE Frost LC Newport PA Hudson N Monoclonal antibody studies of creatine kinase: Antibody-binding sites in the N-terminal region of creatine kinase and effects of antibody onenzyme refolding Biochem. J 1987 248 53 59 3435448 10.1042/bj2480053 PMC1148499

[31] Webb T Jackson PJ Morris GE Protease digestion studies of an equilibrium intermediate in the unfolding of creatine kinase Biochem. J 1997 321 83 88 9003404 10.1042/bj3210083 PMC1218039

[32] Fan YX Zhou JM Kihara H Tsou CL Unfolding and refolding of dimeric creatine kinase equilibrium and kinetic studies Protein Sci 1998 7 2631 2641 9865958 10.1002/pro.5560071217 PMC2143886

[33] Leydier C Clottes E Couthon F Marcillat O Ebel C Vial C Evidence for kinetic intermediate states during the refolding of GdnHCl-denatured MM-creatine kinase. Characterization of a trapped monomeric species Biochemistry 1998 37 17579 17589 9860874 10.1021/bi981828p

[34] Li S Bai JH Park YD Zhou HM Aggregation of creatine kinase during refolding and chaperonin-mediated folding of creatine kinase Int. J. Biochem.: Cell Biol 2001 33 279 286 11311859 10.1016/s1357-2725(01)00003-6

[35] Park YD Ou WB Yu TW Zhou HM Folding pathway for partially folded rabbit muscle creatine kinase Biochem. Cell Biol 2001 79 479 487 11527217

[36] Mazon H Marcillat O Vial C Clottes E Role of C-terminal sequences in the folding of muscle creatine kinase Biochemistry 2002 41 9646 9653 12135386 10.1021/bi025893h

[37] Mazon H Marcillat O Forest E Smith DL Vial C Conformational dynamics of the GdmHCl-induced molten globule state of creatine kinase monitored by hydrogen exchange and mass spectrometry Biochemistry 2004 43 5045 5054 15109263 10.1021/bi049965b

[38] Zhao TJ Ou WB Xie Q Liu Y Yan YB Zhou HM Catalysis of creatine kinase refolding by protein disulfide isomerase involves disulfide cross-link and dimer to tetramer switch J. Biol. Chem 2005 280 13470 13476 15695804 10.1074/jbc.M413882200

[39] Li S Bai JH Park YD Zhou HM Capture of monomeric refolding intermediate of human muscle creatine kinase Protein Sci 2006 15 171 181 16373479 10.1110/ps.051738406 PMC2242377

[40] Ou WB Luo W Park YD Zhou HM Chaperone-like activity of peptidyl-prolyl *cis-trans* isomerase during creatine kinase refolding Protein Sci 2001 10 2346 2353 11604540 10.1110/ps.23301 PMC2374073

[41] Lyubarev AE Kurganov BI Orlov VN Zhou HM Two-state irreversible thermal denaturation of muscle creatine kinase Biophys. Chem 1999 79 199 204 10443013 10.1016/s0301-4622(99)00050-2

[42] Meng FG Hong YK He HW Lyubarev AE Kurganov BI Yan YB Zhou HM Osmophobic effect of glycerol on irreversible thermal denaturation of rabbit creatine kinase Biophys. J 2004 87 2247 2254 15454427 10.1529/biophysj.104.044784 PMC1304650

[43] Gao Y-S Zhao T-J Chen Z Li C Wang Y Yan Y-B Zhou H-M Isoenzyme-specific thermostability of human cytosolic creatine kinase Int. J. Biol. Macromol 2010 47 27 32 20381520 10.1016/j.ijbiomac.2010.03.025

[44] Wallimann T Moser H Eppenberger HM Isozyme-specific localization of M-line bound creatine kinase in myogenic cells J. Muscle Res. Cell Motil 1983 4 429 441 6355174 10.1007/BF00711948

[45] Hornemann T Stolz M Wallimann T Isoenzyme-specific interaction of muscle-type creatine kinase with the sarcomeric M-line is mediated by NH_2_-terminal lysine charge-clamps J. Cell Biol 2000 149 1225 1234 10851020 10.1083/jcb.149.6.1225 PMC2175123

[46] Pelton JT McLean LR Spectroscopic methods for analysis of protein secondary structure Anal. Biochem 2000 277 167 176 10625503 10.1006/abio.1999.4320

[47] Turoverov KK Haitlina SY Pinaev GP Ultra-violet fluorescence of actin. Determination of native actin content in actin preparations FEBS Lett 1976 62 4 6 1248637 10.1016/0014-5793(76)80003-8

[48] Bushmarina NA Kuznetsova IM Biktashev AG Turoverov KK Uversky VN Partially folded conformations in the folding pathway of bovine carbonic anhydrase II: A fluorescence spectroscopic analysis ChemBioChem 2001 2 813 821 11948867 10.1002/1439-7633(20011105)2:11<813::AID-CBIC813>3.0.CO;2-W

[49] Feng S Xu Z Yan Y-B Blocking creatine kinase refolding by trace amounts of copper ions J. Inorg. Biochem 2008 102 928 935 18255152 10.1016/j.jinorgbio.2007.12.013

[50] He H-W Feng S Pang M Zhou H-M Yan Y-B Role of the linker between the N- and Cterminal domains in the stability and folding of rabbit muscle creatine kinase Int. J. Biochem. Cell Biol 2007 39 1816 1827 17616428 10.1016/j.biocel.2007.04.028

[51] Zhao T-J Feng S Wang Y-L Liu Y Luo X-C Zhou H-M Yan Y-B Impact of intrasubunit domain-domain interactions on creatine kinase activity and stability FEBS Lett 2006 580 3835 3840 16797013 10.1016/j.febslet.2006.05.076

[52] Bradford MM A rapid and sensitive method for the quantitation of microgram quantities of protein utilizing the principle of protein-dye binding Anal. Biochem 1976 72 248 254 942051 10.1016/0003-2697(76)90527-3

[53] He G-J Zhang A Liu W-F Cheng Y Yan Y-B Conformational stability and multistate unfolding of poly(A)-specific ribonuclease FEBS J 2009 276 2849 2860 19459940 10.1111/j.1742-4658.2009.07008.x

